# Early Diagnosis of Pancreatic Cancer: The Key for Survival

**DOI:** 10.3390/diagnostics10110869

**Published:** 2020-10-24

**Authors:** Gina Gheorghe, Simona Bungau, Madalina Ilie, Tapan Behl, Cosmin Mihai Vesa, Ciprian Brisc, Nicolae Bacalbasa, Vladiana Turi, Raluca Simona Costache, Camelia Cristina Diaconu

**Affiliations:** 1 Department 5, “Carol Davila” University of Medicine and Pharmacy, 050474 Bucharest, Romania; gheorghe_gina2000@yahoo.com (G.G.); drmadalina@gmail.com (M.I.); ralu_alf@yahoo.com (R.S.C.); 2 Department of Internal Medicine, Clinical Emergency Hospital of Bucharest, 105402 Bucharest, Romania; 3 Department of Pharmacy, Faculty of Medicine and Pharmacy, University of Oradea, 410028 Oradea, Romania; 4 Department of Gastroenterology, Clinical Emergency Hospital of Bucharest, 105402 Bucharest, Romania; 5 Chitkara College of Pharmacy, Chitkara University, Punjab 140401, India; tapanbehl31@gmail.com; 6 Department of Preclinical Disciplines, Faculty of Medicine and Pharmacy, University of Oradea, 410041 Oradea, Romania; v_cosmin_15@yahoo.com; 7 Department of Medical Disciplines, Faculty of Medicine and Pharmacy, University of Oradea, 410041 Oradea, Romania; brisciprian@gmail.com; 8 Department of Surgery, “Ion Cantacuzino” Clinical Hospital, 030167 Bucharest, Romania; nicolae_bacalbasa@yahoo.ro; 9 Department 13, “Carol Davila” University of Medicine and Pharmacy, 050474 Bucharest, Romania; 10 Department of Cardiology, “Victor Babeş” University of Medicine and Pharmacy, 300041 Timisoara, Romania; turi.vladiana@umft.ro; 11 Department of Gastroenterology, “Carol Davila” University Emergency Central Military Hospital, 010825 Bucharest, Romania

**Keywords:** pancreatic cancer, carbohydrate antigen 19-9 (CA19.9), risk factors, early diagnosis, biomarkers

## Abstract

Pancreatic cancer (PC) is one of the most aggressive forms of cancer. Negative prognosis is mainly due to the late diagnosis in advanced stages, when the disease is already therapeutically overcome. Studies in recent years have focused on identifying biomarkers that could play a role in early diagnosis, leading to the improvement of morbidity and mortality. Currently, the only biomarker widely used in the diagnosis of PC is carbohydrate antigen 19-9 (CA19.9), which has, however, more of a prognostic role in the follow-up of postoperative recurrence than a diagnostic role. Other biomarkers, recently identified as the methylation status of ADAMTS1 (A disintegrin and metalloproteinase with thrombospondin motifs 1) and BNC1 (zinc finger protein basonuclin-1) in cell-free deoxyribonucleic acid (DNA), may play a role in the early detection of PC. This review focuses on the diagnosis of PC in its early stages.

## 1. Introduction

Pancreatic cancer (PC) has a low incidence but a high mortality rate [1]. Despite progression in the management of cancers, patients’ survival rate in the case of PC has remained almost unchanged over the past decade [2]. Thus far, PC is the fourth leading cause of cancer death in both genders in the United States of America, after lung, colon, and breast cancer, and the 7th leading cause of cancer-related death all over the world [3,4]. The 5-years survival rate of patients with PC is about 7% [5]. Bad prognosis is, on one hand, due to insidious growth and non-specific symptoms until advanced stages and, on the other hand, to the absence of sensitive and specific methods for early diagnosis [5]. The only therapeutic method with the potential to cure the disease is surgical resection [6]. Unfortunately, only 10–20% of cases are suitable for resection surgery, as over 75% of patients are diagnosed in stages III–IV [5,7].

The disease is characterized by late-onset symptoms, rapid progression, and death [8]. A study showed that approx. 7% of PC patients are diagnosed in a localized stage of the disease [1], which is significantly lower than in other cancers such as breast cancer (61%), colon cancer (40%), lung cancer (16%), ovarian cancer (19%), and prostate cancer (91%) [9]. Late-stage diagnosis is due to non-specific clinical symptoms and signs but also to the reduced incidence of this type of malignant neoplasia. The prevalence of PC in the United States in all ages is about 9 cases/100,000 individuals, rising to 68 cases per 100,000 in individuals older than 55 years [1]. Due to its low prevalence, early detection of PC using screening methods is difficult, with the positive predictive value of any test increasing with the prevalence of the disease [10,11]. From a morpho pathological point of view, more than 95% of pancreatic tumors originate in the exocrine pancreas and less than 5% in the endocrine pancreas [12,13]. Usually, the term “PC” is used for ductal adenocarcinoma, which represents 85–90% of pancreatic tumors [13]. The most used classifications of exocrine pancreatic tumors in the United States are the Armed Forces Institute of Pathology (AFIP) and the World Health Organization (WHO) classifications [13]. These are based on morphological and histological criteria and divide pancreatic tumors into three categories: benign lesions, premalignant lesions and malignant lesions [13,14]. Premalignant tumors have the potential to progress to malignancy. The World Health Organization recommends the use of three degrees of dysplasia to estimate their malignancy potential, such as mild/moderate/severe or low/intermediate/high grade [15]. In the category of malignant tumors, among the most important pathological subtypes are the following [15]:

- ductal adenocarcinoma and its subtypes: 85–90%
- intraductal papillary mucinous neoplasms (IPMN) with an associated invasive carcinoma: 2–3%
- mucinous cystic neoplasms (MCN) with an associated invasive carcinoma: 1%
- acinar cell carcinoma: <1%
- pancreato-blastoma: <1%
- serous cystadenocarcinoma: <1%

Although the incidence of PC did not change significantly in recent years, more frequent use of imaging tests, such as endoscopic ultrasound and helical (spiral) abdominal computed tomography (CT) scans, has led to an increase in the accidental discovery of cystic lesions in the pancreas [15]. Cystic lesions are divided into four main types [10]:

- intraductal papillary mucinous neoplasms (IPMNs)
- mucinous cystic neoplasms (MCNs)
- solid-pseudopapillary neoplasms (SPNs)
- serous cystic neoplasms (SCNs).

IPMN and MCN can progress in time, from lesions with low-grade dysplasia to lesions with high-grade dysplasia and invasive carcinomas [10]. SPNs are low-grade malignant neoplasms, they appear almost always in young women and need to be surgically removed, as opposed to SCN, which is almost always benign and can be monitored clinically, with surgical procedure being recommended when the tumor reaches symptom-inducing sizes [16,17].

Our research presents the main diagnostic tools used for an early diagnosis of PC, such as the presence of certain risk factors, signs and symptoms, laboratory tests, imaging methods, and recent advances of investigation methods. These are of utmost importance, considering the fact that, worldwide, PC is diagnosed in late stages and prognosis is poor. With this in mind, we performed a comprehensive literature search of international data bases (MDPI, Elsevier, Hindawi, Springer, etc.), and 109 papers containing relevant aspects regarding the early diagnosis of PC were retrieved.

## 2. Risk Factors for PC

Various inherited and environmental factors can contribute to and influence the development of PC (Table 1) [10,18]. Approximately 5–10% of PC patients have a family history of this disease [19,20]. There are two categories of patients with a hereditary increased risk of developing PC:

- Patients with associated genetic syndromes (Peutz-Jeghers, ataxia-telangiectasia, Li-Fraumeni); hereditary pancreatitis or ovarian, breast, nonpolyposis colon (Lynch II) cancer; familial atypical multiple mole melanoma syndrome or adenomatous polyposis [19].
- Patients having familial PC history, for which a molecular basis has not yet been discovered [19].

Up to 10% of patients diagnosed with PC have been found to have a genetic susceptibility to the disease [21]. Both inherited and acquired genetic mutations play an important role in the risk of PC. Multiple genes involved in hereditary forms of PC have been established, including genes like BRCA2, CDKN2A, PALB2, STK11, which are high-penetrance genes, and ABO blood group locus, which is a low-penetrance gene [22,23]. Mutations in the BRCA2 gene were identified in about 5–17% of patients with familial PC; they also account for the highest proportion of cases of inherited PC [24]. Mutations in the PALB2 gene, which is a partner and localizer of BRCA2, have been demonstrated to increase the risk of PC as they have been recorded in up to 3% of patients with familial PC [25,26,27,28]. CDKN2A gene mutations are most commonly associated with familial melanoma, while STK11 mutations are seen in patients with Peutz-Jeghers syndrome.

Another group (having a higher risk of PC developing) are those patients with newly diagnosed diabetes. It is estimated that approximately 20–25% of patients with PC develop diabetes 6 to 36 months before the diagnosis of malignancy [29,30,31]. Multiple studies have reported a correlation between type I and II diabetes mellitus and PC [30]. In a study conducted on a group of Italian patients, it was estimated that diabetes was attributable to 9.7% of PCs. Compared with white and black patients, Asian and Hispanic patients diagnosed with diabetes mellitus have a 1.8-fold increase in the risk of developing PC [32,33]. PC risk is inversely proportional to the duration of diabetes. However, a 30% excess risk persists for more than 20 years after diabetes mellitus is diagnosed. Both insulin and oral antidiabetic drugs were correlated with a lower risk of PC. It was shown that, in patients diagnosed with PC and peripheral insulin resistance, the removal of the tumor led to the improvement of glucose metabolism, thus suggesting that altered glucose metabolism may be caused by the tumor [34,35]. A thorough and comprehensive study of the association between diabetes and the risk of PC would be extremely important, particularly in two aspects: the selection of a population at risk for PC and the potential use of recently diagnosed diabetes as a marker of the disease and, in particular, as a specific marker of PC. Other published data proposed a hypothesis that stated precancerous conditions or undiagnosed PC may cause diabetes and insulin resistance, although it was shown that the risk of PC was 1.5- to 2-fold higher in type II diabetes, even when impaired glucose tolerance is detected more than 5 or 10 years before the onset of cancer [36,37,38]. Additional studies are necessary to determine whether diabetes can be used as a marker for prediction of the onset of PC.

An important risk factor for the development of PC is cigarette smoking, as approximately 25% of cases are attributed to this factor [39]. Also, a diet rich in saturated fats, particularly processed or smoked meats, and reduced serum levels of lycopene (a carotenoid present in fruits) and selenium were incriminated in the development of such malignancy [18]. However, the role of dietary supplementation with these micronutrients in reducing the risk of PC remains unclear [40,41].

Smoking is the most significant environmental factor for PC worldwide. There is an inverse association between the risk of PC and the number of cigarettes smoked daily and the duration of smoking. In the last years, the prevalence of cigarette smoking has decreased in some developed countries and increased in others. Moreover, an increase in tobacco smoking has been observed in developing countries and among women. The risk of PC increases in countries that have a high smoking rate. India and China, two of the world’s most densely populated countries, host more smokers than the entire European population [42]. In a study conducted in 2011 in the UK, it was shown that almost 30% of PCs in men and more than 30% in women were associated with tobacco smoking [43].

Regarding alcohol consumption, a correlation between the risk of PC and excessive alcohol use was established, while low and moderate alcohol intake was not associated with risk of developing PC. Concerning the types of alcoholic drinks, a case-control study was performed in 2010 and showed that the consumption of 60 g of liquor per day was correlated with a high risk of PC, while no association with wine or beer was found [44,45].

Obesity is known to be a risk factor for multiple types of cancers, including PC. It was shown that both overweightness and obesity during early adulthood are associated with an increased risk of PC, while obesity during late adulthood was correlated with a decreased survival rate [46,47]. Compared to normal weight people, obese patients had an increased risk of developing PC, according to research conducted by the American Cancer Society [3]. A hypothesis, which stated that both general and abdominal obesity are correlated with an increased risk of PC, was confirmed in a recent study; furthermore, sedentarism, which can lead to fat accumulation, has been associated with increased PC risk [48,49]. High intake of red and processed meats, fried foods and foods containing nitrosamines, may lead to increased PC risk, possibly due to carcinogens and N-nitroso compounds used in the preservation of processed foods. Meta-analysis that included 11 case-control studies showed that PC risk was increased by almost 50% in patients whose diet was high in red meats. In contrast, diets rich in vegetables and fruits have a protective action and determine the reduction of PC risk. A positive correlation has been established between the incidence of PC and a high intake of red or processed meat [50,51,52].

There is also limited data regarding the correlation between the oral microbiome, periodontitis, and PC [53,54]. A study showed that male patients with periodontal disease have a 64% higher risk of developing PC compared with patients without periodontal disease. Another study showed that patients with severe periodontitis have a 400% higher risk of developing PC. The same study demonstrated an association between the increase of anti *Porphyromonas gingivalis* antibodies titer and the gastrointestinal cancer death rate [55,56,57].

On the other hand, there are data in the literature showing that patients with Helicobacter pylori infection have a higher risk of developing PC [55]. One original cohort study, which had initially found a correlation between Helicobacter pylori infection and PC, was updated by increasing the number of patients included in the study. However, no specific correlations with PC risk were identified [58,59]. Another study showed an association between risk of PC and ABO blood type. Helicobacter pylori seropositivity was identified only in patients with non-O blood groups. One of the hypotheses proposed by the authors was the variation of terminal binding antigens in mucins of the gastrointestinal tract depending on blood group. These particularities were associated with Helicobacter pylori’s binding capacity [59,60].

## 3. Clinical Manifestations of PC and Impediments Regarding Early Diagnosis

The initial presentation of patients with PC varies depending on the location of the tumor. In about 60–70% of cases, the tumor is located in the head of the pancreas, whereas in 20–25% of cases it is in the body/tail of the pancreas, and in 5 to 20% of the cases the tumor includes the whole pancreatic body [61]. A study of 185 patients with PC reported the signs and symptoms that these patients have at the time of diagnosis [62]. Table 2 summarizes the main signs and symptoms in pancreatic cancer patients provided by literature data [1,7,61,62].

As Table 2 shows, the most commonly presented symptoms in patients with PC are pain, jaundice, and weight loss [61]. However, a particularity is found in patients with the neoplasm located in the head of the pancreas. These individuals usually present jaundice, weight loss, and steatorrhea [61,62]. Jaundice is a relatively early sign of cephalo-pancreatic cancer, and it also has a prognostic role. Thus, patients who present painless jaundice have been shown to have a more favorable evolution than those presenting obstructive jaundice and pain [61]. Jaundice may occur in patients with tumors in the body/tail of the pancreas later, as a consequence of either primary tumor growth or the appearance of liver metastases [61].

Symptoms of PC include abdominal pain radiating to the back, jaundice, light stool color, loss of appetite, and weight loss. These symptoms are very nonspecific and can be seen in multiple gastrointestinal tract pathologies, such as bile duct tumors and strictures, intestinal ischemia, abdominal aortic aneurysm and ampullary carcinoma. Due to the retroperitoneal position of the pancreas, no diagnostic clinical examination has been proven practical for PC. Moreover, no consensus on the use of diagnostic imaging for early detection of PC has been reached up to the present, as lesions less than 3 cm in size still fail to be detected by most of the imaging techniques used nowadays. PC is characterized by cellular components including fibroblasts, immune cells and stellate cells, and dense desmoplasia with dense fibrous tissue made up of collagen, hyaluronic acid and fibronectin. Tumor cells account for more than 40% of the whole mass, resulting in the dilution of factors secreted by the tumor and making the early detection of PC more difficult [63].

Considering the rarity of PC, for every positive case, 83 false positives will be produced by a biological marker with high sensitivity (100%) and specificity (99%), resulting in unjustified testing [1]. There is a consensus that only high-risk groups of individuals should undergo PC screening [64].

## 4. Diagnosis of PC in Early Stages

The initial evaluation of a patient with suspicion of PC includes serological evaluation and abdominal imaging. Subsequently, depending on the patient’s risk factors, clinical presentation, and initial test results, additional laboratory investigations are performed. Among imaging methods used in the diagnosis and staging of PC are ultrasound, endoscopic ultrasound (EUS), endoscopic retrograde cholangiopancreatography (ERCP), CT, MRI, and PET. These techniques allow for the detection of the pancreatic tumor and the assessment of local or remote dissemination of this malignant disease. They can also help estimate the possibility of surgical resection of the tumor. Another use of these techniques is to monitor the post-operative or post-chemotherapy evolution of the patients and as screening methods for PC in high-risk families [64,65,66,67,68,69,70].

Currently, the most efficient method for certain and early diagnosis of PC is an endoscopic ultrasound (EUS) [71]. This is used to detect and delineate the extent of pancreatic lesions. It also permits us to obtain a biological sample by an FNA biopsy (Fine Needle Aspiration) and microscopic examination. Some of the advantages of using EUS in the diagnosis of PC are:

- The detection of deeply localized tumors that are difficult to identify by transabdominal ultrasound.
- The possibility of obtaining tissue samples by FNA biopsy. The samples then undergo a pathology examination to establish a certain diagnosis. The sensibility of this diagnostic method is approximately 92% [71].
- Higher sensibility when compared with transabdominal US, CT, or MRI in the detection of intraductal papillary mucinous neoplasms < 1 cm in size.
- Higher sensibility when compared with CT in the detection of lymph node metastases and vascular invasion [71].

With the development of new EUS techniques like contrast-upgraded EUS and EUS elastography, the combined use of two or more imaging techniques (CT/US, CT/US/X-ray) can contribute to establishing a certain diagnosis [71,72].

Other imaging means that can be used to ascertain the diagnosis of PC are CT, MRI, and ERCP. These can also be used as screening methods for PC in high-risk patients. Unlike EUS, these techniques have a number of disadvantages like low sensibility in the detection of adenopathies less than 1 cm in size, risk of developing complications like iatrogenic acute pancreatitis (post ERCP), or high cost [73]. Currently, research into PC management is focused on molecular diagnosis as a promising screening method. Thus, studies on the topic suggest the possibility of using circulating tumor cells (CTCs), epigenetic markers or autoantibodies for early diagnosis of this disease, but also for the assessment of prognosis [73].

Table 3 briefly presents the diagnostic algorithm [61,74] of PC, based on the initial clinical presentation. In all patients with jaundice or epigastric pain, serum levels of aminotransferases, alkaline phosphatase, bilirubin, serum lipase, and additionally carbohydrate antigen 19-9 (CA 19-9) should be evaluated. Moreover, in patients with jaundice, it is preferable to perform transabdominal ultrasonography, which can reveal a dilated biliary tract and the location of the obstruction.

However, if there is a high suspicion for choledocholithiasis, ERCP or magnetic resonance cholangiopancreatography (MRCP) can be performed. In patients with epigastric pain and weight loss, an abdominal CT scan is advisable. The last step consists of confirming the diagnosis of this tumor and evaluation of the disease extension by CT scan or magnetic resonance imaging (MRI). Positive diagnosis is established by biopsy and microscopic examination [14,61].

Currently, one of the main targets in terms of management of PC is early detection, thus decreasing the morbidity and mortality of this very aggressive tumor. Iacobuzio-Donahue et al. estimated that there are approximately 10 years from the appearance of the first genetic alterations in patients until the disease is no longer curable. Currently, however, most patients are diagnosed with the disease only in the last two years of this decade.

There are two main categories of methods that can be used for early diagnosis of PC: imaging methods and biomarkers [1]. Ideally, minimally invasive or non-invasive screening methods, such as blood tests that allow early diagnosis, should be preferred. Of the biomarkers, the only one routinely used in the management of PC is carbohydrate antigen 19-9 (CA 19.9) [74]; it has, however, more of a prognostic role, proving to be positive only in 57.1% of patients with PC stage I and 44.1% of patients with stage II [5]. Thus, it may now be used in patients who are suspected of having cancer or pancreatic lesions identified by imaging methods or those with symptoms suggestive of PC, such as obstructive jaundice [75]. It also can be used for monitoring tumor recurrence after treatment [5,76].

## 5. Advances in the Early Diagnosis of PC

**Figure 1:**
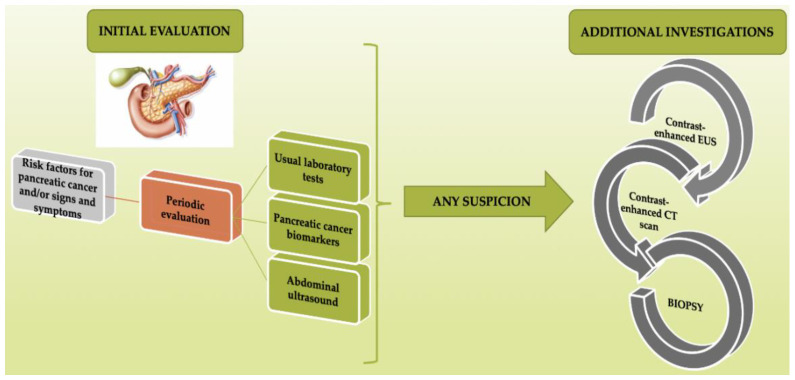
Importance of the early recognition of risk factors and/or signs and symptoms as a starting point of investigation in pancreatic cancer (PC) diagnosis.

The progress made in the molecular diagnosis field, including the detection of circulating malignant cells, circulating proteins, and mucins or miRNAs, may lead to early diagnosis and therefore may improve the prognosis of these patients. An important advantage of molecular techniques is their non-invasiveness, as they only require blood or stool samples. The use of both diagnostic means, molecular-based methods and imaging techniques respectively, showing promising results [77,78,79]. The importance of early recognition of risk factors and/or the signs and symptoms of pancreatic cancer, as a starting point of investigation, is summarized in Figure 1.

### 5.1. Endoscopic Methods

One of the most effective methods currently used for the diagnosis of pancreatic cancer is contrast-enhanced CT. This diagnostic technique has a 90% sensitivity and a 99% specificity. However, contrast-enhanced CT is not widely used because of disadvantages such as high risk of contrast agents related complications, radiation exposure, and high cost. Therefore, contrast-enhanced CT only led to a 6-month increase in the life expectancy of patients diagnosed with PC, with a cost of approximately $2500 per patient [80,81].

Another method used in the diagnosis of PC is MRI, sensitivity and specificity of which are similar to those of contrast-enhanced CT. Due to the high cost and the long period of immobilization it requires, MRI is not broadly utilized [80,81]. Both whole-body CT and MRI examinations can result in false-positive diagnoses. A review showed that out of approximately 90% of cases in which pathological changes suggesting PC were identified, only 2% presented clinical signs [80,81].

Abdominal ultrasound is an imaging technique widely used in the diagnosis of PC, as it is a non-invasive and non-irradiative method. The main disadvantage of abdominal ultrasound is the difficulty of identifying retroperitoneal tumors in an early stage [82,83,84]. The rate of early detection of PC increases when EUS-FNA is utilized. Meta-analysis that included studies conducted between 1995 and 2008 showed that EUS-FNA has an 86.8% sensitivity and a 95.8% specificity in the detection of pancreatic tumors.

Another imaging technique with promising results in PC detection is EUS-elastography. This method involves measurements of tissue elasticity. However, neither EUS nor EUS-elastography are available on a large scale and cannot be considered feasible for the screening of PC [85,86].

### 5.2. Genomic Biomarkers

#### 5.2.1. Epigenetic Biomarkers

In the last years, studies have focused on the identification of non-invasive techniques that could diagnose PC in an early stage. One promising technique is methylation-on-beads technology (MOB). This method involves the analysis of small quantities of DNA found in the peripheral blood. Some biomarkers found in the peripheral blood, with promising roles in the early diagnosis of PC, are the methylation status of ADAMTS1 (A disintegrin and metalloproteinase with thrombospondin motifs 1) and BNC1 (zinc finger protein basonuclin-1) cell-free deoxyribonucleic acid (DNA) [5]. The methylation of DNA is considered to play an important role in the development and progression of cancer [87]. DNA chromatin methylation can alter DNA structure, leading to the suppression of tumor suppressor genes and oncogenes [87]. These genetic changes occur early in the development of several cancers, such as colorectal cancer [88], breast cancer [89,90] and PC [91,92]. A study published in 2019 showed that the methylation status of ADAMTS1 and BNC1 in cell-free DNA is highly sensitive and specific to the early diagnosis of PC, these epidemiological markers varying with tumor stage [5]. Thus, the methylation of ADAMTS1 proved to be positive in 87.5% of patients with stage I cancers, 77.8% of patients with stage IIA, 90% of patients with stage IIB, and 100% of patients with stage III/IV PC [5]. Also, the methylation of BNC1 was positive in 62.5% of patients with stage I cancers, 55.6% of patients with stage IIA, 65% of patients with stage IIB, and 100% of patients with stage III/IV PCs. The use of both biomarkers increased the sensitivity of the diagnostic methods as they were positive in 100% of patients with stage I cancers, 88.9% of patients with stage IIA, 100% of patients with stage IIB, and 100% of patients with stages III/IV of PC [5]. The panel made up of these two biomarkers was also used in patients with chronic pancreatitis, and in 87.5% of cases, both biomarkers were positive.

#### 5.2.2. Circulating Tumor Deoxyribonucleic Acid (ctDNA)

Bettegowda et al. analyzed the utility of ctDNA in the diagnosis of malignant disease. The study involved 640 cancer patients, including patients with pancreatic tumors [79]. ctDNA was identified in more than 75% of the patients with pancreatic, hepatocellular, colorectal, gastroesophageal, ovarian, bladder, breast, melanoma, neck or head tumors and also thyroid, renal or prostate cancer. Regarding early-stage cancer, ctDNA was identified in 73% of patients with colorectal disease, 57% of patients with gastroesophageal cancer, 48% of patients with PC, and 50% of patients with breast adenocarcinoma [79]. An important aspect to note is that ctDNA was also identified in patients without any circulating tumor cells [79].

#### 5.2.3. MicroRNAs (miRNAs)

MicroRNAs are small, non-encoding RNAs of 18–22 nucleotides that are involved in regulating gene expression and play an important role in some cellular mechanisms, including tumorigenesis [93,94,95]. A study that analyzed pancreatic tissue samples obtained by FNA and a surgical biopsy demonstrated different miRNA profiles in patients with pancreatic ductal adenocarcinoma (PDAC) and intraductal papillary mucinous neoplasm (IPMN), compared with healthy individuals [93]. 607 deregulated miRNAs were found in PDAC and 396 miRNAs were found in IPMN, using next-generation sequencing (NGS). In both lesions, quantitative reverse transcriptase PCR validated 30 overexpressed miRNAs [93]. The possibility of identifying these changes in tissue samples obtained by FNA or plasma makes them potential biomarkers for the early detection of pancreatic cancer.

#### 5.2.4. Stool-Based Tests

Other non-invasive tests that can be used as screening methods are stool-based tests. Cologuard (indicated for colorectal cancer screening) is a kit that includes a molecular analysis for DNA mutations (such as KRAS mutation), methylation biomarkers such as BMP3 and NDRG4 methylation, and an immunochemical assay for human hemoglobin. The reference gene used for the quantification of total human DNA found in every sample is beta-actin [1,96]. For a stool biomarker to play a role in the diagnosis of pancreatic cancer, it must be well expressed in the pancreatic tissue and poorly expressed in the epithelium of the digestive tract. According to existing data, among the markers identified in the stool that may contribute to the early diagnosis of pancreatic cancer are the mutant KRAS gene and methylated BMP3. The detection of these biomarkers may be influenced by age, not by gender or smoking history. Other stool biomarkers which may be useful in the early detection of pancreatic cancer are NDRG4 or UCHL1. Future studies are needed to clarify the role of these biomarkers in the diagnosis of PC [97].

### 5.3. Proteomic Biomarkers

Tumor derived proteins that can be detected in blood, pancreatic juice, tumor tissue or cell lines may contribute to the early diagnosis of PC [98]. Table 4 shows the potential proteomic biomarkers that may be useful in the early diagnosis of PC, based on the analyzed biological samples, and Table 5 presents some proteomic biomarkers and their utility in PC diagnosis.

## 6. Conclusions

PC is one of the utmost aggressive cancers, with a high mortality rate. Negative prognosis is mainly due to late diagnosis in the advanced stages of the disease, when it is already therapeutically overcome. Due to the low incidence, a screening strategy in these patients is not feasible in terms of cost-effectiveness. However, recent studies have shown the benefits of using screening for PC in high-risk patients, such as those with familial history of PC, those who have specific mutations known to be associated with increased risk of PC, or those with a long history of smoking [60,106,107]. Early diagnosis is extremely important because it can increase the percentage of candidates for surgical resection of the tumor. Given that chemotherapy has shown only moderate benefits in these patients, surgery remains the only potentially curative method [108,109]. One cost-effective method for the early diagnosis of PC is the use of biomarkers tested in the peripheral blood. So far, the only used biomarker for PC is CA 19.9, which has more of a prognostic role and in tracking tumor recurrence after treatment, rather than a diagnostic role. Methylation of ADAMTS1 and BNC1 showed a certain sensitivity and specificity for the early diagnosis of this neoplasia. These data highlight the need for future studies to pursue the discovery of methods for the early diagnosis of PC. Used on a large scale, these could lead to an improvement in the survival rates of patients with this malignancy.

## Figures and Tables

**Table 1:** Risk factors involved in the development of pancreatic cancer.

Intrinsic Risk Factors	Extrinsic Risk Factors
Hereditary	Cigarette smoking
AB0 blood group	Obesity and physical inactivity
Cystic fibrosis	Diet
Nonhereditary chronic pancreatitis	Coffee and alcohol consumption
Diabetes mellitus and insulin resistance	Aspirin and nonsteroidal anti-inflammatory drugs use
Pancreatic cysts	Helicobacter pylori infection
	Hepatitis B and C virus (HBV and HCV) infections

**Table 2:** The most relevant signs and symptoms present at diagnosis in patients with pancreatic cancer.

Symptoms	Signs
Asthenia	Jaundice
Weight loss	Hepatomegaly
Anorexia	Right upper quadrant mass
Abdominal pain	Cachexia
Epigastric pain	Courvoisier’s sign
Dark urine	Epigastric mass
Jaundice	Ascites
Nausea	
Back pain	
Diarrhea	
Vomiting	
Steatorrhea	
Thrombophlebitis	

**Table 3:** The diagnostic algorithm in pancreatic cancer.

Initial Testing		2nd Step		3rd Step
**Jaundice or epigastric pain**	Serum aminotransferases, alkaline phosphatase, bilirubin, serum lipase, and additionally carbohydrate antigen 19-9 (CA 19-9). These laboratory tests raise suspicion of an upper abdominal cavity pathology, CA19-9 being the most widely used biomarker for PC, with 44.2% sensitivity and 84.8% specificity [74].	**Jaundice**	Transabdominal US or ERCP or MRCP in patients with high suspicion for choledocholithiasis.	1. To confirm the presence of the mass and to assess disease extent: CT scan or MRI. 2. Positive diagnosis: BIOPSY–percutaneous biopsy, endoscopic ultrasound-guided biopsy.
		**Epigastric pain and weight loss**	Abdominal CT	

Legend: CA 19-9–carbohydrate antigen 19-9; CT–Computed tomography; ERCP–Endoscopic retrograde cholangiopancreatography; MRCP–Magnetic resonance cholangiopancreatography; MRI–magnetic resonance imaging; PC–pancreatic cancer; US–ultrasound.

**Table 4:** Potential proteomic biomarkers for the early diagnosis of pancreatic cancer.

Proteomic Biomarkers			
In Tissue	In Cell Lines	In Pancreatic Juice	In Serum/Plasma
Actinin-4 Annexin A2 Bcl-2 Cathepsin D CD34 CEACAM5 COX-2 Galectin-1 H1.3 hENT1 IGFBP2 IGFBP3 Integrin 1 Ki-67 Laminin β1 LGALS3BP MUC5AC P27 P53 Plasminogen S100A4 Survivin TGF-β1 VEGF	ApoE CD9 Fibronectin receptor Perlecan S100A6 S100A8 S100A9 SDF4 SMAD4	A1BG Caldecrin DJ-1 FGB Lithostathine I α MMP-9 L1CAM Plasminogen S100A8 S100A9	CA 19-9 DKK1 Exosomal glypican-1 HSP-27 IL-11 MIC-1 Xylitol+ 1,5-anhydro-D-glucitol + histidine + inositol CA 19-9 + MUC5AC CA 19-9 + CA 242 CA 19-9 + IGF-1 + albumin CA 19-9 + CEA + CA 125 + CA 242 CA 19-9 + 5MC + H2AZ + H2A1.1 + H3K4Me2 CA 19-9 + CEA + HGF + OPN + ctDNA CA 19-9 + THBS2

Legend: Bcl-2–B-cell lymphoma 2; CD34–hematopoietic progenitor cell antigen CD34; CEACAM5–Carcinoembryonic Antigen-Related Cell Adhesion Molecule 5; COX-2–cyclooxigenase-2; H1.3–Histone 1.3; hENT1–human equilibrative nucleoside transporter 1; IGFBP2–Insulin Like Growth Factor Binding Protein 2; IGFBP3–Insulin Like Growth Factor Binding Protein 3; Ki-67–nuclear protein associated with cellular proliferation; LGALS3BP–lectin galactoside-binding soluble 3 binding protein; MUC5AC–a protein coding gene, oligomeric mucus/gel-forming; P27–protein P27; P53–tumor protein P53; S100A4–S100 calcium-binding protein A4; TGF-β1–transforming growth factor beta 1; VEGF–vascular endothelial growth factor; ApoE–apolipoprotein E; CD9–protein coding gene CD9; S100A6–S100 calcium-binding protein A6; S100A8-S100 calcium-binding protein A8; SDF4–stromal cell derived factor 4; SMAD4–SMAD family member 4; A1BG–alpha-1-B glycoprotein; DJ-1–protein deglycase DJ-1; MMP-9–matrix metalloproteinase 9; L1CAM–L1 Cell Adhesion Molecule; CA 19-9–cancer antigen 19-9; DKK1–Dickkopf WNT Signaling Pathway Inhibitor 1; HSP-27–heat shock protein 27; IL-11–interleukin 11; MIC-1–macrophage inhibitory cytokine 1; CA242–carbohydrate antigen 242; IGF-1–insulin-like growth factor-1; CEA–carcinoembrionic antigen; CA 125–cancer antigen 125; 5MC–5-Methylcytosine; H2AZ–Histone H2AZ; H2A1.1–Histone H2A1.1; H3K4Me2-Histone H3 dimethylated at lysine 4; HGF–hepatocyte growth factor; OPN–osteopontin; ctDNA–Circulating tumour deoxyribonucleic acid; THBS2–Thrombospondin 2.

**Table 5:** Biomarkers used in PC diagnosis.

Biomarker(s)	Sample Origin	Main Findings and Observations–Usefulness in PC Diagnosis	Ref.
C4BPA	Serum	Higher sensitivity than CA19-9, particularly in the early stages of PDAC, also higher specificity for PDAC.	[99]
CPA4	Serum + tissue biopsy	Very well expressed in the tissues and serum of patients with PC, useful in staging the disease because it is associated with lymph node metastasis.	[100]
GPC1	Serum	Correlation with tumor staging, can be identified in circulating exosome of patients with PC, but it is not found in healthy subjects.	[101]
MUC5AC	Serum + tissue biopsy	In combination with CA 19-9, it can be useful in improving sensitivity; it is undetectable in healthy pancreas tissue.	[102]
OPNT + TIMP-1	Serum	Combined with CA 19-9, it can improve the sensitivity for PC, therefore being useful in early detection; it is also undetectable in healthy individuals.	[103]
PFAA profile	Serum	Strong correlations with disease stage, can distinguish healthy individuals from ones suffering from PC.	[104]
LYVE1, REG1A, TFF1	Urine	High accuracy, of over 90%. PC early stage is detectable using this biomarkers panel.	[105]

Legend: C4BPA–C4b-binding protein α-chain; CPA4–Carboxypeptidase A4; ctDNA–Circulating tumour deoxyribonucleic acid; GPC1–Glypican-1; MUC5AC–a protein coding gene, oligomeric mucus/gel-forming; OPN–Osteopontin; PC–Pancreatic cancer; PDAC–Pancreatic ductal adenocarcinoma; PFAA–Plasma free amino acid; TIMP-1–Tissue inhibitor of metalloproteinase 1; and LYVE1, REG1A, TFF1,–Tri-marker panel.
